# The effect of deviations in sintering temperature on the translucency and color of multi-layered zirconia

**DOI:** 10.1186/s12903-024-04243-4

**Published:** 2024-04-18

**Authors:** Fan Yang, Luyao Zhang, Minghui Yang, Jianfeng Chen, Wenzhong Xing

**Affiliations:** 1 Graduate School of Dalian Medical University, Dalian, China; 2 Dental Technology Center, Dalian Stomatological Hospital, Dalian, China; 3 Department of Stomatology, The First Affiliated Hospital of Dalian Medical University, Dalian, China; 4 Department of Prosthodontics, Dalian Stomatological Hospital, Dalian, Liaoning 116021 PR China

**Keywords:** Multi-layered zirconia, Sintering temperature, Color, Translucency

## Abstract

**Object:**

This study aimed to investigate the changes in the translucency and color of four different multi-layered zirconia materials when the sintering temperature were inaccurate.

**Materials and methods:**

Two hundred zirconia samples (11 × 11 × 1.0 mm) of four multi-layered zirconia, Upcera TT-GT (UG), Upcera TT-ML (UM), Cercon xt ML (CX), and Lava Esthetic (LE), were divided into five subgroups according to the sintering temperature: L1 (5% lower temperature), L2 (2.5% lower temperature), R (recommended sintering temperature), H2 (2.5% higher temperature), H1 (5% higher temperature). After sintering, color coordinates were measured. Then the translucency parameter (TP) values, and the color differences (between the inaccurate sintering temperature and the recommended temperature) of each zirconia specimen were calculated. Statistical analysis was performed by using three-way ANOVA tests, the one-way ANOVA, and Tukey’s post hoc test.

**Results:**

Three-way ANOVA results showed that material type, sintering temperature, specimen section, and their interactions significantly influenced the TP values (except for the interactions of specimen section and sintering temperature) (*P* < .05). TP values of zirconia specimens were significantly different in the inaccurate sintering temperatures (*P* < .05), except for the cervical and body sections of UG group (*P* > .05). Compared with recommended sintering temperature, higher sintering temperature caused higher TP values for CX, but lower for LE. Three-way ANOVA results showed that material type, sintering temperature, and their interactions significantly influenced the ∆*E*_00_ values (*P* < .05). There were no significant differences in ∆*E*_00_ values of UM and CX groups at different inaccurate sintering temperatures, and were clinical imperception (except for UM-L1) (∆*E*_00_ < 1.25). ∆*E*_00_ values of all zirconia specimens showed clinically acceptable (∆*E*_00_ < 2.23).

**Conclusion:**

The deviations in sintering temperature significantly influenced the translucency and color of tested multi-layered zirconia. The trends of translucency in the multi-layered zirconia depended on material type and the color changes of all zirconia materials were clinically acceptable at inaccurate sintering temperatures.

## Background

Zirconia restorations were popularly applied in prosthetic clinics due to their promising biocompatibility, chemical resistance and exceptional mechanical properties. However, the esthetic restoration of zirconia ceramics has always been a challenge due to their white opaque appearance [1].

Zirconia is a polycrystalline material that exists in nature in three forms: monoclinic (m-phase), tetragonal (t-phase), and cubic (c-phase). Among these forms, the c-phase is optically isotropic and no-birefringent, leading to greater light transmission and consequently increased translucency [2]. Y_2_O_3_ is the widely used stabilizing oxide to make zirconia stabilize to c-phase at room temperature [3]. Higher yttria content (4–6 mol%) increases the content of cubic phase in zirconia, improving the translucency of the zirconia material [2].

At present, the ultra-translucent zirconia materials with 5 mol% yttria content (5Y-PSZ, 5 mol% yttria-partially stabilized zirconia) are used for restoration in the anterior regions. The cubic phase becomes the main phase of 5Y-PSZ, thus exhibiting similar translucency to lithium-disilicate, which is very good in esthetic and is stronger than glass ceramic [3, 4]. In particular, multi-layered zirconia systems have been developed to further improve the esthetic properties of dental restorations and mimic the shade gradient like natural teeth. Multi-layered zirconia presents an incisal section like enamel, a body section like dentin, and a cervical section with a masking effect, thus becoming a possible restoration solution, especially in anterior regions [5].

To realize optimal restoration results, zirconia ceramic is colored by adding pigments to zirconia powder or immersing the uncolored zirconia in coloring liquids [6]. According to the instruction of the manufacturer, in multi-layered zirconia, the gradient color outcome is achieved by adding a small amount of shading elements (e.g. iron and rare earth elements) to the white zirconia base material, gradually increased from the incisal section to the cervical section. After sintering, these elements are built into the zirconia crystals and provide the desired gradient shade effect.

The translucency and color of zirconia materials are crucial keys to achieving a natural character, simulating the appearance of natural tooth structure for restorations, and selecting material, especially in esthetic regions [7, 8]. Translucency Parameter (TP) and the color difference (∆*E*_00_) have been proven to be reliable indicators of optical properties of zirconia [9, 10]. The optical properties of zirconia restoration are influenced by several factors, such as the original shade of zirconia ceramics or stains [11], the thickness, microstructure [12] and sintering process [13]. Furthermore, the microstructure and crystalline content of zirconia is determined by the sintering procedure and temperature [8, 12, 14]. Particularly, sintering temperature is a crucial parameter, directly impacting the growth of zirconia particles, grain size, sintered density and porosity [14, 15]. So it is crucial to maintain the sintering temperature under control. Haag et al. [16] measured the actual sintering temperature of 20 commercial dental furnaces to identify the accuracy of sintering temperatures in dental furnaces. The results showed that there was always an inaccuracy of +/−5% between the setting temperature and the actual temperature varied from the furnace brands and conditions [16]. A previous study found that 5% deviations from the recommended sintering temperature influenced the transmittance of zirconia with different yttria content [17]. Nevertheless, there were limited researches on the effect of the inaccuracy sintering temperature on the translucency and color of different brands of multi-layered zirconia material.

The purpose of this study was to investigate the effect of 2.5% and 5% sintering temperature deviations compared to recommended sintering temperatures on the translucency and color of different multi-layered zirconia. The null hypothesis was that sintering temperature deviations would not affect the optical properties of different multi-layered zirconia.

## Materials and methods

### Fabrication of specimens

The disk-shaped samples (200 pieces at 14 × 14 × 1.3 mm) were produced from four multi-layered zirconia (UG: Upcera TT-GT, UM: Upcera TT-ML, CX: Cercon xt ML, LE: Lava™ Esthetic Fluorescent Full-Contour Zirconia) in A2 shade (Table 1). All samples were prepared by using a low-speed diamond cutter and diamond saw blades (SYJ-150, Shenyang Kejing Automation Equipment Co Ltd., Shenyang, China) under dry conditions, then finely ground with 600 grits of sandpapers (3M ESPE, St. Paul, MN, USA) to the final dimensions.

**Table 1:** Code, manufacturers, composition and lot numbers of materials used

Material	Code	Manufacturer	Composition	Lot Number
TT-GT, A2	UG	Upcera Dental Technology, Shenzhen, China	ZrO_2_ + HfO_2_ + Y_2_O_3_ > 96.5%; 5.8-9.7%Y_2_O_3_; Al_2_O_3_ < 0.5%, Fe_2_O_3_ < 0.5%, Er_2_O_3_ < 2.0%, Other oxides < 0.5%	-
TT-ML, A2	UM	Upcera Dental Technology, Shenzhen, China	ZrO_2_ + HfO_2_ + Y_2_O_3_ > 96.5%; 5.8-9.7%Y_2_O_3_; Al_2_O_3_ < 0.5%, Fe_2_O_3_ < 0.5%, Er_2_O_3_ < 2.0%, Other oxides < 0.5%	-
Cercon xt ML, A2	CX	Dentsply Sirona, Charlotte, NC, USA	ZrO_2_; 9%Y_2_O_3_; HfO_2_ < 3%; Al_2_O_3_, SiO_2_, other oxides < 2%	18044511
Lava™ Esthetic Fluorescent Full-Contour Zirconia, A2	LE	3M, St. Paul, MN, USA	ZrO_2_; 5 mol% Y_2_O_3_	7484966

### Sintering process

Fifty discs of each material were divided into five groups (*n* = 10) according to the sintering temperatures. All the specimens were sintered with the furnace (inLab Profire, Dentsply Sirona, Bensheim, Germany) according to their sintering protocols respectively. Five different temperatures (H1, H2, R, L2, L1) were carried out in the sintering protocols of each material. H1: 5% higher than recommended temperature, H2: 2.5% higher than recommended temperature, R: manufacturer’s recommended sintering temperature, L2: 2.5% lower than recommended temperature, L1: 5% lower than recommended temperature. The temperatures for CX and LE groups (H1: 1575 °C H2: 1535 °C R: 1500 °C L2: 1465 °C L1: 1425 °C) were higher than UG (H1: 1555 °C H2: 1515 °C R: 1480 °C L2: 1445 °C L1: 1405 °C) and UM groups (H1: 1522 °C H2: 1486 °C R: 1450 °C L2: 1420 °C L1: 1375 °C). A block of temperature measurement (Referthermo, Japan Fine Ceramics Center, Nagoya, Japan) was placed in the furnace in each sintering protocol to ensure the actual sintering temperature inside the furnace. The setting and actual temperatures for each sintering protocol are given in Table 2.

**Table 2:** The temperature of setting and actual in the sintering furnace respectively

Material		Sintering temperature (°C)				
		H1	H2	R	L2	L1
UG	ST	1555	1515	1480	1445	1405
	AT	1545	1505	1470	1443	1407
UM	ST	1522	1486	1450	1420	1375
	AT	1512	1478	1447	1424	1387
CX and LE	ST	1575	1535	1500	1465	1425
	AT	1569	1526	1494	1461	1427

*ST means setting temperature, and AT means the actual temperature in furnace

UG and UM groups were started at room temperature, sintered at the rate of 8 °C per minute heating to 1150 °C holding for 30 min. Then heated to 1300 °C at the rate of 2 °C per minute, and subsequently, sintered to maximum temperature at the rate of 4 °C per minute and held for 120 min. After that, cooled them at the rate of 8 °C per minute to 800 °C, finally cooled naturally to room temperature.

CX group was started at room temperature, heated at the rate of 3 °C per minute to 500 °C, then heated to 1200 °C at the rate of 8 °C per minute, and held for 30 min, then heated to 1300 °C at the rate of 2 °C per minute. Subsequently, heated them to the maximum temperature at the rate of 4 °C per minute holding 120 min. After that, cooled them at the rate of 8 °C per minute to 800 °C, finally cooled naturally to room temperature.

LE group was started at room temperature, with a 22 °C per minute rate heated to 800 °C, then heated to maximum temperature at the rate of 10 °C per minute. After 120-minute step time, the temperature was decreased to 800 °C at a 15 °C per minute cooling rate, finally cooled to 250 °C at a rate of 20 °C per minute.

After sintering programs, the sample surfaces of color measurement were serially polished using 800, 1200, 1500, 2000, and 4000 grits sandpapers to achieve the ideal smooth surface. The final dimensions (11 × 11 × 1.0 ± 0.02 mm) of sintered specimens were measured with a digital micrometer (Mitutoyo Corp, Kawasaki, Japan) after ultrasonically cleaning in distilled water for 10 min.

### Processing of composite resin background disks

Light-curing resin of A2 shade (Z350^XT^, Dentine, 3M ESPE, St Paul, MN, USA) was filled in silicon rubber (Silagum Putty Soft, DMG, Hamburg, Germany) mold (14 mm×14 mm×4.5 mm) and pressed with a glass plate to ensure a flat surface. Then cured using a light-polymerizing unit (Mini LED, Satelec, Merignac, France) for 40 s on both sides. The color measurement surface of composite resin was polished with 600 grit wet silicon-carbide paper, adjusting the thickness at 4.0 ± 0.02 mm.

### Color measurement

The CIE L*a*b* (L*, brightness; a*, red-green value; and b*, yellow-blue value) values of zirconia specimens were measured using a spectrophotometer (Crystaleye, Olympus, Tokyo, Japan) which used 7 light-emitting diodes (LEDs) as the illumination source with 45/0-degree geometry [18]. For each sample, three measurements were taken and their average was recorded. Translucency was determined by measuring CIE L*a*b* values for all samples against a standard white background (CIE L*=90.33 a*=-0.53 b*=0.79) and black background (CIE L*=20.53 a*=-0.74 b*=-1.00). Then, specimens were placed over the A2 shade composite resin background (CIE L*=74.28 a*=1.54 b*=18.82) to measure their CIE L*a*b* values. Distilled water was put between the zirconia specimen and the background to acquire optical contact during the measurement process. The color difference (∆*E*_00_) was determined between the specimens sintered at recommended temperature and inaccurate temperature. All measurements were made on the cervical, body, and incisal areas of each specimen, and after three measurements, recording the average.

### Translucency, color difference and chroma calculation

The TP values were determined with the following formula:

TP = [(L*_B_ - L*_W_)^2^ + (a*_B_ - a*_W_)^2^ + (b*_B_ - b*_W_)^2^]^1/2^

L*, a* and b* refers to brightness, red-green, and yellow-blue coordinates [19]. Where B and W are the color coordinates over a standard black and white backing. The TP value ranges from 0 to 100, with lower values indicating materials with lower translucency and higher values indicating materials with higher translucency.

The chroma of specimens was calculated with the following formula:

C*_ab_ = (a*^2^ + b*^2^)^1/2^

The color differences (∆*E*_00_) were calculated based on the following formula:

$$\begin{array}{l}\Delta {E_{{\bf{00}}}} = \\\sqrt {{{\left( {\frac{{\Delta {\bf{L}}\prime }}{{{K_L}{S_L}}}} \right)}^2} + {{\left( {\frac{{\Delta {\bf{C}}\prime }}{{{K_C}{S_C}}}} \right)}^2} + {{\left( {\frac{{\Delta {\bf{H}}\prime }}{{{K_H}{S_H}}}} \right)}^2} + {R_T}\left( {\frac{{\Delta {\bf{C}}\prime }}{{{K_C}{S_C}}}} \right)\left( {\frac{{\Delta {\bf{H}}\prime }}{{{K_H}{S_H}}}} \right)} \end{array}$$

Where ΔL', ΔC' and ΔH' represent the differences in lightness, chroma and hue between the two specimens respectively; S_L_, S_C_, and S_H_ are weighting functions that adjust the total color difference based on changes in the position of the color difference pair in the L*, a* and b* coordinates; K_L_, K_C_ and K_H_ are parameter factors, and were entirely set to 1 in this experiment [20]. Mean ∆*E*_00_ values below 1.25 were assumed “clinically imperceptible”, while mean ∆*E*_00_ values above 2.23 were assumed “clinically unacceptable” [21].

### Statistical analysis

The data analysis was performed by using a statistical software program (IBM SPSS Statistics, v25.0; IBM Corp., Armonk, NY, USA). Three-way and One-way ANOVAs were performed to analyze the effect of sintering temperature, the material type, and specimen section on the translucency and color difference followed by the Tukey post hoc tests [22] at a significance level of *P* < .05.

## Results

According to the setting temperature, the actual sintering temperatures were shown in Table 2.

## Variations in TP values at different sintering temperatures

The three-way ANOVA test showed that material type, sintering temperature, specimen section, and their interactions significantly influenced the TP values (except for the interactions of specimen section and sintering temperature) (*P* < .05) (Table 3).

**Table 3:** Three-way ANOVA analysis of variance of translucency parameter results

Source	Type III Sum of Squares	df	Mean Square	F	*P*
material type	2137.424	3	712.475	1279.801	< 0.001*
sintering temperature	278.705	4	69.676	125.158	< 0.001*
specimen section	61.666	2	30.833	55.384	< 0.001*
material type×sintering temperature	1426.806	12	118.901	213.578	< 0.001*
material type×specimen section	222.362	6	37.06	66.57	< 0.001*
sintering temperature×specimen section	7.842	8	0.98	1.761	0.082
material type×sintering temperature×specimen section	24.069	24	1.003	1.801	0.012*
Error	300.622	540	0.557		
Total	458638.080	600			
Corrected total	4459.496	599			

*Statistically significant difference at *P* < .05

**Fig. 1:**
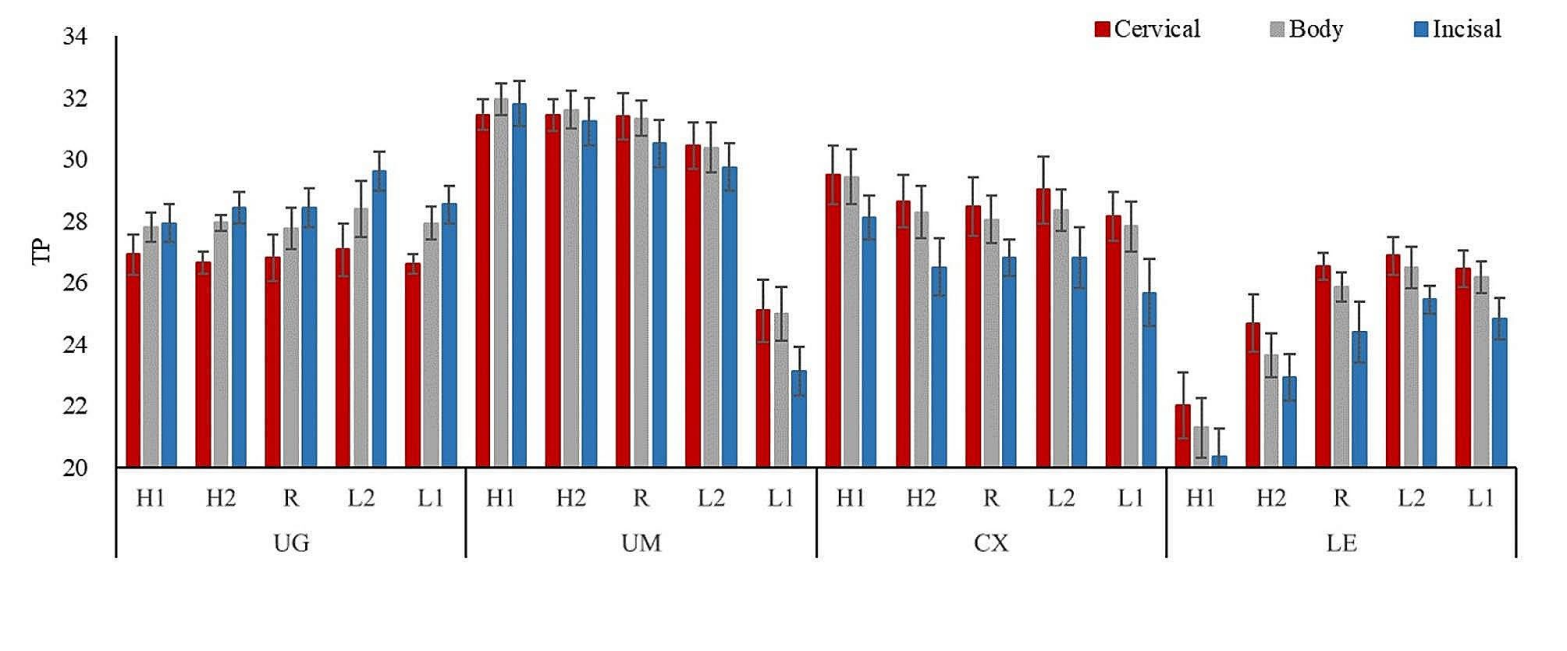
The mean values of TP for zirconia materials at different sintering temperatures in three sections

One-way ANOVA results showed that sintering temperature significantly affected the TP values of specimens except for the cervical and body sections of UG group (*P* < .05) (Table 4). When the sintering temperature was higher than recommended, there were no significant differences in TP values of zirconia specimens in each group except for the body and incisal sections of CX group, the incisal section of UM group, and all sections in LE group. When the sintering temperature was lower than recommended temperature, there were no significant differences in TP values of zirconia specimens in each group except for the whole UM group and the incisal sections of UG, CX and LE groups. The translucency of the three sections of LE and UM group decreased obviously when the sintering temperature was H1 and L1, respectively. TP values were similar in UG group or UM group when the sintering temperature was H1, H2 and R (Fig. 1).

**Table 4:** Mean and SD values of translucency of materials in three sections at different sintering temperatures

Material	Section	Sintering temperature					F	*P*
		H1	H2	R	L2	L1		
UG	Cervical	26.91 ± 0.66	26.65 ± 0.36	26.82 ± 0.75^B^	27.07 ± 0.85	26.60 ± 0.32	0.947	0.446
	Body	27.80 ± 0.48	27.95 ± 0.26	27.76 ± 0.68^A^	28.39 ± 0.90	27.93 ± 0.52	1.678	0.172
	Incisal	27.93 ± 0.63^b^	28.42 ± 0.52^b^	28.42 ± 0.64^A,b^	29.62 ± 0.63^a^	28.55 ± 0.61^b^	10.584	< 0.001
UM	Cervical	31.44 ± 0.49^a^	31.44 ± 0.52^a^	31.39 ± 0.74^A,a^	30.45 ± 0.76^b^	25.09 ± 1.01^c^	142.966	< 0.001
	Body	31.94 ± 0.52^a^	31.60 ± 0.62^a^	31.33 ± 0.56^A,a^	30.38 ± 0.80^b^	24.97 ± 0.87^c^	177.074	< 0.001
	Incisal	31.81 ± 0.71^a^	31.22 ± 0.77^a,b^	30.51 ± 0.76^B,b,c^	29.75 ± 0.79^c^	23.13 ± 0.78^d^	213.791	< 0.001
CX	Cervical	29.48 ± 0.95^a^	28.63 ± 0.85^a,b^	28.46 ± 0.95^A,a,b^	29.00 ± 1.09^a,b^	28.15 ± 0.80^b^	3.020	0.027
	Body	29.43 ± 0.90^a^	28.28 ± 0.86^b^	28.04 ± 0.77^A,b^	28.34 ± 0.68^b^	27.82 ± 0.80^b^	5.953	0.001
	Incisal	28.10 ± 0.72^a^	26.51 ± 0.94^c^	26.82 ± 0.59^B,b^	26.81 ± 0.98^b^	25.67 ± 1.08^c^	9.888	< 0.001
LE	Cervical	22.02 ± 1.06^c^	24.68 ± 0.93^b^	26.53 ± 0.43^A,a^	26.87 ± 0.62^a^	26.46 ± 0.60^a^	70.769	< 0.001
	Body	21.30 ± 0.97^c^	23.65 ± 0.71^b^	25.85 ± 0.47^A,a^	26.48 ± 0.67^a^	26.17 ± 0.51^a^	102.171	< 0.001
	Incisal	20.34 ± 0.94^d^	22.92 ± 0.76^c^	24.40 ± 0.99^B,b^	25.44 ± 0.45^a^	24.84 ± 0.67^a,b^	67.390	< 0.001

*Groups with different superscript lower case letters (a, b) have significant differences in row (Tukey’s test). Groups with different superscript upper case letters (A, B) have significant differences in column (Tukey’s test)

When UM, CX and LE groups were sintered at recommended sintering temperature, TP values of tested zirconia specimens showed a decreasing tendency from the cervical to the incisal section, and there were no significant differences between cervical and body sections (*P* > .05). TP values of UG group showed an increasing tendency from the cervical to incisal section, and there was no significant difference in body and incisal sections of UG group (*P* > .05) (Table 4).

### Variations in ∆*E*_00_ values at different sintering temperatures

The three-way ANOVA test showed that material types, sintering temperatures, and their interactions significantly influenced the ∆*E*_00_ values (*P* < .05) (Table 5). The interactions of material type and specimen section significantly affected the ∆*E*_00_ values (*P* < .05), and the specimen section did not (*P* > .05) (Table 5).

**Table 5:** Three-way ANOVA analysis of variance of color difference between inaccuracy temperatures and recommended sintering temperature

Source	Type III Sum of Squares	*df*	Mean Square	F	*P*
material type	8.956	3	2.985	12.884	< 0.001*
sintering temperature	26.282	3	8.761	37.807	< 0.001*
specimen section	0.121	2	0.060	0.260	0.771
material type×sintering temperature	28.310	9	3.146	13.574	< 0.001*
material type×specimen section	3.611	6	0.602	2.597	0.018*
sintering temperature×specimen section	0.267	6	0.044	0.192	0.979
material type ×sintering temperature×specimen section	3.698	18	0.205	0.886	0.596
Error	100.106	432	0.232		
Total	775.204	480			
Corrected total	171.351	479			

*Statistically significant difference at *P* < .05

**Fig. 2:**
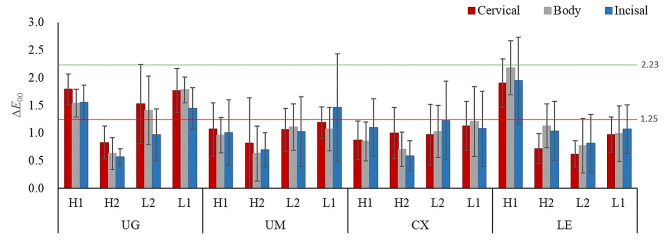
The mean values of ∆*E*_00_ of UG, UM, CX and LE in three sections

The results of one-way ANOVA showed that sintering temperatures significantly affected the ∆*E*_00_ values of specimens (except for UM and CX groups) (*P* < .05). ∆*E*_00_ values of all tested specimens sintered between R and other inaccurate temperatures varied from 0.57 to 2.18, which were clinically acceptable (∆*E*_00_ < 2.23) (Table 6). ∆*E*_00_ values of UG group (except ∆*E*_00 R−L2_ in incisal section and ∆*E*_00 R−H2_ in all sections), and ∆*E*_00 R−H1_ of LE group in all sections were above the perceptibility threshold (∆*E*_00_ > 1.25). ∆*E*_00_ values of CX and UM groups (except ∆*E*_00 R−L1_ of UM group in incisal section) were all below the limit of perceptibility threshold (Fig. 2).

**Table 6:** Mean and SD values of color difference of zirconia materials in three sections

Material	Section	Sintering temperature				F	*P*
		∆*E*_00 R−H1_	∆*E*_00 R−H2_	∆*E*_00 R−L2_	∆*E*_00 R−L1_		
UG	Cervical	1.79 ± 0.28^a^	0.83 ± 0.30^b^	1.53 ± 0.71^a^	1.77 ± 0.40^a^	9.811	< 0.001
	Body	1.54 ± 0.25^a^	0.63 ± 0.29^b^	1.41 ± 0.62^a^	1.78 ± 0.23^a^	17.348	< 0.001
	Incisal	1.56 ± 0.31^a^	0.57 ± 0.15^b^	0.97 ± 0.47^b^	1.45 ± 0.37^a^	17.618	< 0.001
UM	Cervical	1.07 ± 0.48	0.82 ± 0.82	1.06 ± 0.39	1.19 ± 0.28	0.823	0.490
	Body	0.96 ± 0.32	0.63 ± 0.50	1.11 ± 0.42	1.07 ± 0.39	2.759	0.056
	Incisal	1.01 ± 0.59	0.70 ± 0.31	1.03 ± 0.63	1.46 ± 0.97	2.197	0.105
CX	Cervical	0.87 ± 0.35	1.00 ± 0.46^A^	0.97 ± 0.55	1.13 ± 0.44	0.561	0.644
	Body	0.85 ± 0.35	0.71 ± 0.31^A,B^	1.03 ± 0.47	1.21 ± 0.63	2.342	0.089
	Incisal	1.10 ± 0.52	0.59 ± 0.27^B^	1.23 ± 0.71	1.08 ± 0.68	2.386	0.085
LE	Cervical	1.90 ± 0.44^a^	0.72 ± 0.27^b^	0.62 ± 0.24^b^	0.97 ± 0.32^b^	31.806	< 0.001
	Body	2.18 ± 0.49^a^	1.13 ± 0.40^b^	0.77 ± 0.49^b^	0.99 ± 0.50^b^	17.633	< 0.001
	Incisal	1.95 ± 0.78^a^	1.04 ± 0.53^b^	0.82 ± 0.52^b^	1.07 ± 0.44^b^	7.293	0.001

*Groups with different superscript lower case letters (a, b) have significant differences in row (Tukey’s test). Groups with different superscript upper case letters (A, B) have significant differences in column (Tukey’s test)

### Variations in L* and C*_ab_ values at different sintering temperatures

**Fig. 3:**
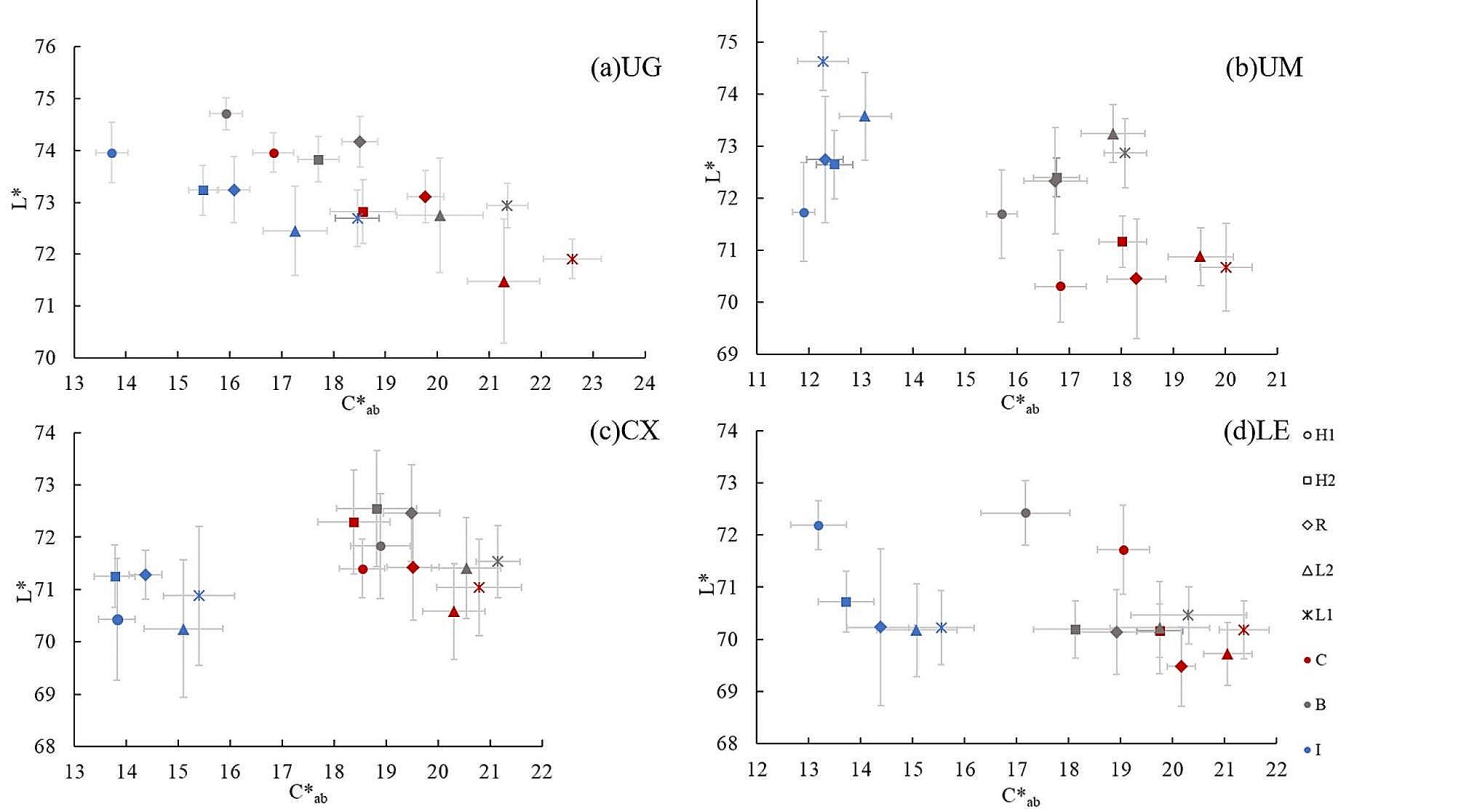
The distribution of L* and C*_ab_ of materials at different sintering temperatures in three sections

Distributions of the L* and C*_ab_ values of zirconia specimens in different sintering temperatures were presented in Fig. 3. L* values decreased in UG group and increased in the incisal section of UM group with the sintering temperature reducing (Fig. 3a, c). When the sintering temperature was 5% higher than recommended, the L* values of UG and LE groups increased. The C*_ab_ values of all specimens decreased when the sintering temperature was higher than recommended. C*_ab_ values increased from the incisal to cervical section, and L* values were minor changes.

## Discussion

The null hypothesis was rejected because the results of this study revealed that the translucency and color difference were influenced by sintering temperature deviations.

Sintering procedures directly affects the grain size, porosity, yttria distribution and the content of the cubic phase in zirconia specimens [14, 23]. In the current study, the variations in sintering temperature caused the different tendencies of translucency change in zirconia specimens. TP values of CX (except for cervical section) and the incisal section of UM groups specimens sintered at H1 were higher than those sintered at R. This finding was in line with other previous studies in which the TP values of zirconia materials increased with the sintering temperature increased [14, 24, 25]. The phenomenon could be attributed to the fact that higher sintering temperature increases the grain size, reduces the grain boundary density and refraction, thus increasing the translucency of zirconia [8]. On the contrary, TP values of LE group significantly decreased at H1 and H2 temperatures compared with the recommended sintering temperature. Attachoo et al. [25] reported that the translucency of zirconia (Ceramill® Zolid classic) decreased when the sintering temperature (1550 °C) was higher than regular temperature (1350 °C). This result may be associated with the microcrack nucleation in grain boundary, which adversely affecting the light scattering.

The TP values decreased when the sintering temperature was lower than recommended temperature, zirconia became opaque. Especially in UM group, TP values of specimens sintered at L1 and L2 were significantly lower than those sintered at R. Similar results were found by Vult et al. [17], who investigated the effect of 5% sintering temperature deviations on the optical properties of zirconia with different yttria content. They observed that the transmittance values of tested zirconia (3YSB-E®, 3YSBC® and Zpex®smile, colorless) sintered at T_L_ (5% lower than recommended) were lower than at T_R_ (recommended sintering temperature) and T_H_ (5% higher than recommended). Lower sintering temperature led to reduced grain size and increased grain boundary, which therefore influenced the light scattering and diminished the translucency of zirconia [8, 17].

The multi-layered zirconia has a layered structure designed to mimic the color gradient observed in natural teeth. In the present study, UM, CX and LE groups showed a slight decrease in translucency from the cervical to incisal section, while UG group showed an increased tendency. The results were consistent with the results of Uasuwan et al. [26], who found Cercon xt ML in A2 shade showed a slight decrease in TP values from the cervical to incisal section. This could make the incisal section less impacted by the oral black background and improve shade reproducibility in multi-layered zirconia restorations. Based on the results of the color parameters, except for CX group, the cervical section showed the least brightness compared with the body and incisal sections in other groups. The distributions of C*_ab_ showed an obvious decreased tendency from the cervical to incisal section in the chromatic of zirconia specimens [27]. However, there is no difference in the tetragonal, cubic phase content and grain size in each layer [5]. The optical properties of different layers were probably attributed to the zirconia composition and the discrepancy in pigmentation among different layers [27, 28].

The deviations in sintering temperature had different effects on the color results of multi-layered zirconia, but they were all clinically acceptable (∆*E*_00_ < 2.23), regardless of sintering temperature deviations and specimen sections. Cardoso et al. [29] compared the optical properties of Prettau Anterior zirconia sintered at 1450 °C and 1600 °C temperatures, and observed that the color difference was perceptible but acceptable (0.81 <∆*E*_00_ < 1.77). In the present study, the ∆*E*_00_ of UG group (except for ∆*E*_00 R−H2_, and ∆*E*_00 R−L2_ in incisal section), ∆*E*_00 R−L1_ of UM group in incisal section and ∆*E*_00 R−H1_ of LE group were clinical perceptible (∆*E*_00_ > 1.25). This was because higher and lower sintering temperatures would influence the sintered density of zirconia specimens, leading to the changes in pores and crystal arrangement. The changes in the light transmission and reflection ultimately influence the color results of zirconia [30]. Thus, the deviations in sintering temperature greatly affected the color outcome of UG group. Higher sintering temperature (H1) led to significant color changes in LE group.

The color parameters of zirconia specimens were influenced by inaccurate sintering temperatures. When the sintering temperature was inaccurate, the tendency of C*_ab_ values of all specimens was the same irrespective of material type, while the tendency of L* values varied depending on material type. As the sintering temperature decreased, all zirconia specimens increased in chroma, and the brightness increased in the incisal sections of UM group, while decreasing in UG group.

The limitations of this study were that the zirconia samples were flat, and the multi-layered zirconia samples tested in the study had similar yttria content with only 1 shade (A2). Furthermore, surface roughness, X-ray diffractometry, and elemental analysis of the zirconia specimens at inaccurate sintering temperatures were not conducted in the present study. Future research should consider combining the mentioned factors that can affect the optical and mechanical properties of multi-layered zirconia materials.

## Conclusion

Based on the settings and the results, this study indicated that the deviations in sintering temperature influenced the translucency and color of tested multi-layered zirconia, which mainly depended on the types of multi-layered zirconia. The inaccurate sintering temperatures led to different trends of translucency in different types of multi-layered zirconia. Too low sintering temperature (L1) led to a significant reduction in translucency. The deviations in sintering temperature greatly influenced the color outcome of some zirconia materials, while the color results of all zirconia materials were clinically acceptable.

## Data Availability

The datasets generated and analyzed during the current study are available from the corresponding author on reasonable request.

## Abbreviations

TP: Translucency parameter
